# The Cumulative Effect of Gene-Gene and Gene-Environment Interactions on the Risk of Prostate Cancer in Chinese Men

**DOI:** 10.3390/ijerph13020162

**Published:** 2016-01-27

**Authors:** Ming Liu, Xiaohong Shi, Fan Yang, Jianye Wang, Yong Xu, Dong Wei, Kuo Yang, Yaoguang Zhang, Xin Wang, Siying Liang, Xin Chen, Liang Sun, Xiaoquan Zhu, Chengxiao Zhao, Ling Zhu, Lei Tang, Chenguang Zheng, Ze Yang

**Affiliations:** 1 Department of Cell Biology and Genetics, School of Basic Medical Science, Shanxi Medical University, Taiyuan 030001, China; lium0421@163.com (M.L.); 13934662586@outlook.com (C.Z.); 2 The Key Laboratory of Geriatrics, Beijing Hospital & Beijing Institute of Geriatrics, Ministry of Health, Beijing 100730, China; sxh_cnu@163.com (X.S.); zuoyeyf@163.com (F.Y.); baobaocathh@163.com (S.L.); sunbmu@foxmail.com (L.S.); zhuxqyy@gmail.com (X.Z.); tanglei1974@hotmail.com (L.T.); 3 Graduate School, Peking Union Medical College and Chinese Academy of Medical Sciences, Beijing 100005, China; 4 Department of Urology and Beijing Hospital, Chinese Ministry of Health, Beijing 100730, China: dongwei63@126.com (D.W.); zhangyaoguang2005@hotmail.com (Y.Z.); wangxingemini@163.com (X.W.); 18600405631@163.com (X.C.); 5 Department of Urology, The Second Hospital of Tianjin Medical University, Tianjin 300211, China; xuyong8816@sina.com (Y.X.); ykuoster@126.com (K.Y.); 6 Medical Examination Centre, Beijing Hospital, Ministry of Health, Beijing 100730, China; zhuling8899@hotmail.com; 7 Guangxi Zhuang Autonomous Region Women and Children Care Hospital, Nanning, Guangxi 530003, China; oushangenetics2012@163.com

**Keywords:** gene-gene interaction, gene-environment interaction, prostate cancer, single nucleotide polymorphism

## Abstract

Prostate cancer (PCa) is a multifactorial disease involving complex genetic and environmental factors interactions. Gene-gene and gene-environment interactions associated with PCa in Chinese men are less studied. We explored the association between 36 SNPs and PCa in 574 subjects from northern China. Body mass index (BMI), smoking, and alcohol consumption were determined through self-administered questionnaires in 134 PCa patients. Then gene-gene and gene-environment interactions among the PCa-associated SNPs were analyzed using the generalized multifactor dimensionality reduction (GMDR) and logistic regression methods. Allelic and genotypic association analyses showed that six variants were associated with PCa and the cumulative effect suggested men who carried any combination of 1, 2, or ≥3 risk genotypes had a gradually increased PCa risk (odds ratios (ORs) = 1.79–4.41). GMDR analysis identified the best gene-gene interaction model with scores of 10 for both the cross-validation consistency and sign tests. For gene-environment interactions, rs6983561 CC and rs16901966 GG in individuals with a BMI ≥ 28 had ORs of 7.66 (*p* = 0.032) and 5.33 (*p* = 0.046), respectively. rs7679673 CC + CA and rs12653946 TT in individuals that smoked had ORs of 2.77 (*p* = 0.007) and 3.11 (*p* = 0.024), respectively. rs7679673 CC in individuals that consumed alcohol had an OR of 4.37 (*p* = 0.041). These results suggest that polymorphisms, either individually or by interacting with other genes or environmental factors, contribute to an increased risk of PCa.

## 1. Introduction

Prostate cancer (PCa) is a complex multifactorial disease. A twin study suggested that genetic factors may explain 42% of the etiological risk of PCa [1]. Genome-wide association studies (GWAS) identified over 70 PCa susceptibility variants, providing evidence of genetic susceptibility in the development of PCa. However, these polymorphic loci were common variants, mostly with low penetrance [2]. The odds ratios (ORs) of these PCa-associated single nucleotide polymorphisms (SNPs) were modest (1.02–1.66) and no one locus contributed highly to the risk of PCa [3].

Gene-gene interactions play a role in potential mechanisms of the missing heritability in human genetics, and research has identified the phenomenon in PCa. For example, Tao et al. identified 1325 pairs of SNP–SNP interactions with a P cutoff of 1.0 × 10^-8^ in 1176 PCa cases and 1101 control subjects from the National Cancer Institute Cancer Genetic Markers of Susceptibility study, although no SNP-SNP interaction reached a genome-wide significance level of 4.4 × 10^-13^ [4]. A study by Ciampa et al. showed that two biologically interesting interactions, one between rs748120 of NR2C2 and subregions of 8q24 and that between rs4810671 of SULF2 and both JAZF1 and HNF1B, were associated with PCa [5]. In cases of Italian heredo-familial PCa, VDR1 T/T genotypes coupled with the VDR2 T/T genotype exhibited a five-fold higher probability of PCa [6]. Additionally, some studies showed that the cumulative effect of such interactions could increase the ORs of PCa. Specifically, the risk of PCa in men with six or more risk alleles was higher than in men with two or fewer risk alleles (OR = 6.22) [7]. In a Swedish population, the OR for PCa was 9.46 in men who had five or more SNPs associated with PCa, compared with men without any of these SNPs [8].

Environmental factors play a significant role and perhaps can modify the genetic risk of PCa [9]. Body mass index (BMI), smoking, and alcohol consumption are PCa-related environmental factors that affect the risk of this disease. A meta-analysis of prospective studies indicated that obesity may have a dual effect on the risk of PCa: a decreased risk for localized PCa and an increased risk for advanced PCa [10]. Smoking cessation can reduce the risk of developing PCa [11]. Alcohol consumption is related to an increased risk of PCa in a Chinese population [12].

Despite identifying dozens of PCa risk variants using GWAS, as well as the emergence of evidence of environmental risk factors that contribute to PCa, the effect of gene-gene and gene-environment interactions on the risk of PCa is largely unknown. In the current study, 36 SNPs were selected from a GWAS and used to estimate their association with PCa in 286 cases and 288 control subjects. We determined the gene-gene interactions and cumulative effects between the confirmed PCa risk SNPs. Finally, the gene-environment interactions in 134 PCa patients were analyzed to identify the combination of factors yielding the greatest risk of PCa in Chinese men.

## 2. Materials and Methods

### 2.1. Study Population

This was a case-control study including 286 PCa patients and 288 healthy geographically matched controls. All subjects were unrelated Northern Han Chinese men, and were permanent residents of Beijing or Tianjin (Jingjin area). Detailed inclusion criteria of cases and controls were described previously [13]. The age at diagnosis, the Gleason score, tumour stage, serum PSA levels of PCa patients were obtained and aggressive PCa was defined as tumours with a PSA level > 20 ng/mL, and/or a Gleason score ≥8 or higher, and/or a pathological stage ≥III [14]. This study was approved by the ethics committee of the two participating hospitals (ethical approval number: 2013BJYYEC-047-01), and informed consent was obtained from all study participants.

Height and body weight were measured in 134 Beijing PCa cases, and information about smoking and alcohol consumption were collected. BMI was calculated using the individual’s height and weight (kilograms per square meter), and categorized according to standard cut-off points for the Chinese population (underweight <18.5, normal weight 18.5–24.0, overweight 24.0–28.0, and obese ≥28.0) [15]. In our study, all patients were classified into normal, overweight, or obese groups. 

Questionnaire data collected about smoking included addiction (current, past, or never smoked), age of smoking initiation, years of smoking, number of cigarettes smoked per day, and years since quitting smoking. Individuals smoking every day, or some days, were classified as current smokers. Those who responded “not at all” were classified as never smokers, and individuals with a smoking history but who had quit were classified as past smokers [16]. Because of the small sample size in our study, the number of smoking classifications was reduced. Past smokers who had smoked more than 100 cigarettes in their lifetime were classified as current smokers and those who had smoked fewer than 100 cigarettes in their lifetime were classified as never smokers. 

Individuals that consumed alcohol were grouped into four categories: non-drinking, drinking on special occasions only, often drinking, and drinking every day [17]. Non-drinking and drinking occasionally individuals were classified as “seldom”. Drinking every day and drinking often were classified as “often”.

### 2.2. Selection of SNPs for Genotyping

Thirty-six PCa-associated SNPs, identified in a GWAS from 2007 to 2010 and a Multiethnic Cohort study of a functional locus, were selected for genotyping [18,19,20,21,22,23,24,25,26,27]. Blood genomic DNA was extracted and the selected loci were genotyped as described previously [13]. rs11986220 was genotyped by sequencing while the other 36 SNPs were determined by using polymerase chain reaction-high resolution melting curves (PCR-HRM) of small amplicons and sequencing methods. Briefly, a final reaction volume of 10 µL included 1 µL of 1× LC-green PLUS fluorescent dye and 0.05 µL of each pair of low and high temperature calibrators (10 pmol/µL). After the PCR reaction, products were transferred into matching 96-well plates to be genotyped automatically and verified manually using a Lightscanner TMHR-I 96 (Idaho Technology, Inc., Salt Lake City, UT, USA). To validate the accuracy of genotyping, about 10% of the samples were selected randomly for duplicate analysis. In addition, five samples were selected randomly from the three different verified genotypes of each risk variant to be sequenced (Beijing Genomics Institute, Beijing, China) to confirm the genotyping results. All primers used for both PCR-HRM and PCR sequencing were designed using Oligo (version 6.0; Molecular Biology Insights, Inc., Cascade, CO, USA). Tables S1 and S2 list the information about the primers that were used.

### 2.3. Gene-Gene Interaction Analyses

The Generalized Multifactor Dimensionality Reduction (GMDR) (GMDR Software, Beta version 0.7; Department of Biostatistics, University of Alabama, Birmingham, AL, USA) method, with age adjustment, was used to determine gene-gene interactions between PCa-associated SNPs [28,29]. Ten-fold cross-validation (CV) was set when running the software. The age-adjusted model with the smallest prediction error (the greatest testing balanced accuracy), the highest CV consistency, and *p* < 0.05 in the sign test was selected as the best n-loci model. In this study, we analyzed the interaction among six SNPs associated with a risk for PCa (rs16901966, rs11986220, rs1447295, and rs10090154 at 8q24; rs1983891 at FOXP4; rs339331 at GPRC6A) by using a GMDR model that adjusted for age. Entropy-based interaction dendrograms were built by using Multifactor Dimensionality Reduction (MDR) to better confirm and visualize the interaction models identified by GMDR [30].

### 2.4. Cumulative Effect Analysis

Based on the results of the association analyses between 36 SNPs and PCa, the cumulative effect was determined among five confirmed PCa risk loci, excluding rs11986220, which is linked with rs10090154. Combined with genotyping data, samples that carried none, one, two, or more than two of the risk genotypes were labeled 0, 1, 2, and ≥3, respectively. Risk genotypes were defined from the genetic model analyses of rs1983891 TT/TC, rs339331 TT, rs16901966 GG, rs11986220 AA/AT, rs1447295 AA/AC, and rs10090154 TC/TT. ORs and 95% confidence intervals (CIs) were calculated to compare the frequency of risk genotypes between PCa cases and controls. The logistic regression method was used to calculate the ORs and 95% CIs (or the age-adjusted ORs and 95% CIs).

### 2.5. Gene-Environment Interaction Analyses

A logistic regression test was used for the gene-environment interaction analyses. For gene-BMI interactions, BMI < 24.0, BMI = 24.0–28.0, and BMI ≥ 28.0 were defined as 1, 2, and 3, respectively. For gene-smoking interactions, never smokers and current smokers were defined as 1 and 2, respectively. For gene-drinking interactions, seldom and often were defined as 1 and 2, respectively. During multinomial logistic regression calculations, independent genotypes of the 36 SNPs were categorized as “dependent” and environmental factors were categorized as “factor”, while age was selected as “covariate” when adjusting the regression. In general, the non-risk genotypes and those in an unexposed environment (BMI < 24.0, never smoking, and seldom drinking) were used as the control group, and compared with the risk genotype and those in an exposed environment (BMI = 24.0–28.0, BMI ≥ 28.0, current smoking, and often drinking).

### 2.6. Statistical Analyses

Pearson’s χ^2^ was used to test the Hardy-Weinberg equilibrium (HWE) for each SNP separately among the control subjects. Logistic regression was used to estimate the unadjusted and age-adjusted ORs and 95% CIs for each risk allele (designated as “1”) versus each non-risk allele (designated as “2”). Genotypes 11, 12, and 22 represented risk homozygotes, risk heterozygotes, and non-risk homozygotes, respectively. When ORs and 95% CIs were calculated by logistic regression in the different models, genotypes 11 + 12 were designated as “1” and genotype 22 was designated as “2” in a dominant mode, and genotype 11 was designated as “1” and genotypes 12 + 22 were designated as “2” in a recessive mode. Logistic regression analyses were also used to estimate ORs and 95% CIs, and age adjusted ORs and 95% CIs, of cumulative effects. Statistical analyses were performed using the Statistical Package for the Social Sciences software package (version 16.0; SPSS, Inc., Chicago, IL, USA), and *p* < 0.05 was considered significant.

## 3. Results

### 3.1. Demographics

Characteristics of the 286 PCa cases and 288 controls are shown in Table 1. The mean age ± SD of cases and controls were 72.3 ± 7.5 years (range from 46 to 93) and 70.5 ± 7.6 years (range from 59 to 89), respectively (*p* = 0.005). Within the 134 PCa cases, BMI < 24.0, BMI = 24–28, and BMI ≥ 28 were 57 (42.5%), 61 (45.5%), and 16 (11.9%) cases, respectively. Never and current smokers were 73 (54.7%) and 61 (45.3%) cases, respectively. Seldom and often drinkers were 116 (86.6%) and 18 (13.4%), respectively.

**Table 1:** Selected demographic characteristics of study subjects.

Characteristics	Cases	Control	*p*
Number of subjects	286	288	-
Age, years (mean [SD])	72.30 (7.458)	70.47 (7.604)	0.005
Range	46–93	59–89	-
PSA (ng/mL)			
Range	0.15–1338	0–4	–
<10	102	–	–
10–20	40	–	–
>20	78	–	–
Gleason score	146	–	–
4–6	51	–	–
7	50	–	–
8–10	45	–	–
Tumor stage	138	–	–
I	9	–	–
II	69	–	–
III	46	–	–
IV	14	–	–
Aggressiveness	163	–	–
Nonaggressive PCa	48	–	–
Aggressive PCa	115	–	–
A positive family history of PCa	8	–	–
BMI	134	–	–
<24.0	57	–	–
24–28	61	–	–
≥28	16	–	–
Smoking	134	–	–
Never	73	–	–
Current	61	–	–
Drinking	134	–	–
Seldom	116	–	–
Often	18	–	–

### 3.2. Allelic and Genotypic Associations with PCa

Table 2 displays the allelic frequencies of the 36 SNPs. Analyses of the allelic frequencies of PCa cases and controls showed that rs1465618-A, rs1983891-T, rs339331-T, rs16901966-G, rs1447295-A, rs11986220-A, and rs10090154-T SNPs were associated with a 28%–57% increase in the risk of PCa (age-adjusted OR 1.28–1.57; *p* = 0.050–0.005).

Genotypic frequencies of all 36 SNPs among control subjects, except SNP rs2710646 (*p* < 0.05), did not deviate from the HWE (*p* > 0.05) (Table S3). The genotype distribution in genetic models showed that rs1983891, rs10086908, rs1447295, rs11986220, and rs10090154 were associated with a risk of PCa in the dominant model (age-adjusted ORs 1.44–1.71; *p* = 0.034–0.002). rs339331, rs1016343, rs16901966, rs7127900, and rs4430796 were associated with a risk of PCa in the recessive model (age-adjusted ORs 1.51–2.26; *p* = 0.025–0.007) (Table 3). Sequencing results were consistent with the genotypes of variants identified by HRM.

Based on these results, only rs16901966, rs11986220, rs1447295, and rs10090154 at 8q24, rs1983891 at FOXP4, and rs339331 at RFX6 of the 36 SNPs examined, were associated with PCa in the allele and genotype association analyses.

**Table 2:** Association analysis between the alleles of 36 SNPs and PCa in Chinese men.

Region		Alleles	RAF		Unadjusted Allelic OR		Age-adjusted Allelic OR		Power
	refSNP ID	(1/2) *	Case	Control					
			1	1	OR (95% CI)	*p*	OR (95% CI)	*p*	
EHBP1	rs721048	A/G	0.038	0.024	1.20 (0.65–2.19)	0.563	1.74 (0.84–3.62)	0.138	0.278
EHBP1	rs2710646	A/C	0.042	0.035	1.61 (0.78–3.32)	0.201	1.30 (0.71–2.39)	0.402	0.380
THADA	rs1465618	A/G	0.694	0.646	1.25 (0.97–1.60)	0.085	1.31 (1.02–1.69)	**0.034**	0.408
ITGA6	rs12621278	A/G	0.720	0.719	1.01 (0.78–1.30)	0.960	1.02 (0.78–1.32)	0.899	0.050
C2orf43	rs13385191	G/A	0.472	0.444	1.12 (0.89–1.41)	0.341	1.11 (0.88–1.40)	0.384	0.159
3p12	rs2660753	A/G	0.338	0.330	1.04 (0.80–1.34)	0.780	1.03 (0.80–1.34)	0.814	0.060
3q21	rs10934853	A/C	0.423	0.432	0.97 (0.76–1.22)	0.772	0.97 (0.76–1.22)	0.767	0.060
PDLIM5	rs17021918	C/T	0.650	0.667	0.93 (0.73–1.18)	0.535	0.93 (0.73–1.19)	0.573	0.093
TET2	rs7679673	C/A	0.225	0.210	1.10 (0.82–1.46)	0.525	1.08 (0.81–1.44)	0.599	0.094
5p15	rs12653946	T/C	0.431	0.398	1.15 (0.91–1.45)	0.252	1.12 (0.88–1.42)	0.344	0.206
SLC22A3	rs9364554	T/C	0.327	0.342	0.94 (0.73–1.21)	0.619	0.93 (0.72–1.20)	0.596	0.084
FOXP4	rs1983891	T/C	0.355	0.303	1.27 (1.00–1.63)	0.055	1.28 (1.00–1.65)	**0.050**	0.313
GPRC6A/RFX6	rs339331	T/C	0.690	0.620	1.40 (1.10–1.79)	**0.007**	1.35 (1.06–1.74)	**0.017**	0.703
LMTK2	rs6465657	C/T	0.878	0.863	1.15 (0.81–1.63)	0.426	1.11 (0.78–1.57)	0.557	0.117
NKX3-1	rs1512268	A/G	0.340	0.312	1.14 (0.89–1.46)	0.304	1.17 (0.91–1.51)	0.212	0.173
8q24 (Block1)	rs12543663	C/A	0.101	0.084	1.23 (0.82–1.83)	0.318	1.26 (0.84–1.9)	0.257	0.169
8q24 (Block1)	rs10086908	T/C	0.786	0.803	0.89 (0.65–1.22)	0.473	0.89 (0.65–1.23)	0.499	0.110
8q24 (Block2/Region2)	rs1016343	T/C	0.429	0.384	1.20 (0.95–1.52)	0.124	1.21 (0.95–1.54)	0.115	0.342
8q24 (Block2/Region2)	rs13252298	G/A	0.285	0.287	1.01 (0.78–1.31)	0.954	1.00 (0.77–1.31)	0.988	0.051
8q24 (Block2/Region2)	rs6983561	C/A	0.300	0.254	1.26 (0.97–1.63)	0.086	1.27 (0.98–1.66)	0.073	0.414
8q24 (Block2/Region2)	rs16901966	G/A	0.299	0.244	1.32 (1.02–1.72)	**0.037**	1.33 (1.02–1.73)	**0.035**	0.554
8q24 (Block3/Region3)	rs16902094	G/A	0.288	0.265	1.12 (0.86–1.46)	0.388	1.14 (0.88–1.49)	0.327	0.140
8q24 (Block3/Region3)	rs445114	T/C	0.546	0.542	1.07 (0.71–1.60)	0.757	1.05 (0.70–1.58)	0.829	0.052
8q24 (Block3/Region5)	rs620861	C/T	0.559	0.562	0.99 (0.79–1.26)	0.958	1.00 (0.79–1.27)	0.991	0.051
8q24 (Block4/Region3)	rs6983267	G/T	0.463	0.424	1.17 (0.92–1.48)	0.196	1.15 (0.90–1.45)	0.261	0.265
8q24 (Block5/Region1)	rs1447295	A/C	0.224	0.164	1.47 (1.09–1.98)	**0.011**	1.49 (1.10–2.01)	**0.010**	0.729
8q24 (Block 5/Region 1)	rs11986220	A/T	0.211	0.145	1.59 (1.16–2.16)	**0.004**	1.57 (1.14–2.15)	**0.005**	0.832
8q24 (Block5/Region1)	rs10090154	T/C	0.208	0.145	1.55 (1.14–2.12)	**0.006**	1.53 (1.12–2.10)	**0.008**	0.800
8q24 (Block5/Region1)	rs7837688	T/C	0.203	0.163	1.3 (0.96–1.77)	0.088	1.28 (0.94–1.75)	0.112	0.418
MSMB	rs10993994	T/C	0.522	0.491	1.13 (0.88–1.46)	0.328	1.12 (0.87–1.44)	0.385	0.183
11p15	rs7127900	G/A	0.847	0.900	0.61 (0.41–0.92)	**0.018**	0.60 (0.40–0.90)	**0.013**	0.470
11q13	rs7931342	G/T	0.260	0.283	0.89 (0.69–1.16)	0.388	0.90 (0.69–1.17)	0.436	0.141
13q22	rs9600079	T/G	0.464	0.436	1.12 (0.89–1.41)	0.341	1.09 (0.86–1.38)	0.469	0.159
HNF1B	rs4430796	A/G	0.764	0.709	1.33 (0.95–1.86)	0.099	1.31 (0.93–1.83)	0.123	0.562
17q24	rs1859962	G/T	0.453	0.409	1.20 (0.94–1.52)	0.150	1.21 (0.94–1.54)	0.134	0.325
KLK2/KLK3	rs2735839	G/A	0.579	0.607	0.89 (0.70–1.13)	0.335	0.88 (0.69–1.12)	0.290	0.162

Note: * Risk alleles are listed first (1) in the allele column. *p* < 0.05 are in bold.

**Table 3:** Association analysis between the different genetic models of 36 SNPs and PCa in Chinese men.

		Unadjusted Genotypic OR				Age-Adjusted Genotypic OR			
	Additive Model	Dominant Model		Recessive Model		Dominant Model		Recessive Model	
refSNP ID	(df = 2)	(11 + 12 *vs.* 22)		(11 *vs.* 12 + 22)		(11 + 12 *vs.* 22)		(11 *vs.* 12 + 22)	
	*p*	OR (95%CI)	*p*	OR (95% CI)	*p*	OR (95% CI)	*p*	OR (95% CI)	*p*
rs721048	0.310	1.46 (0.69–3.09)	0.327	-	-	1.57 (0.74–3.36)	0.242	-	-
rs2710646	0.784	1.24 (0.64–2.40)	0.524	-	-	1.34 (0.69–2.6)	0.396	-	-
rs1465618	0.480	1.53 (0.94–2.5)	0.085	1.36 (0.97–1.89)	0.072	1.55 (0.95–2.53)	0.081	1.32 (0.95–1.85)	0.103
rs12621278	0.949	0.94 (0.52–1.71)	0.839	1.03 (0.74–1.43)	0.861	0.95 (0.52–1.75)	0.877	0.99 (0.71–1.38)	0.939
rs13385191	0.293	1.02 (0.71–1.46)	0.906	1.37 (0.91–2.04)	0.131	1.01 (0.70–1.45)	0.955	1.35 (0.90–2.03)	0.149
rs2660753	0.915	1.02 (0.72–1.43)	0.917	1.12 (0.66–1.90)	0.676	1.00 (0.71–1.42)	0.983	1.13 (0.66–1.93)	0.652
rs10934853	0.849	0.92 (0.65–1.30)	0.615	1.02 (0.67–1.55)	0.931	0.90 (0.63–1.27)	0.539	1.05 (0.68–1.60)	0.833
rs17021918	0.289	1.15 (0.70–1.90)	0.578	0.82 (0.59–1.14)	0.228	1.12 (0.68–1.86)	0.649	0.83 (0.60–1.17)	0.288
rs7679673	0.179	0.99 (0.71–1.40)	0.967	2.00 (0.91–4.38)	0.084	0.98 (0.69–1.38)	0.885	1.96 (0.89–4.33)	0.095
rs12653946	0.210	0.97 (0.69–1.38)	0.881	1.35 (0.87–2.09)	0.187	0.94 (0.66–1.34)	0.725	1.33 (0.85–2.07)	0.216
rs9364554	0.369	0.84 (0.59–1.17)	0.299	1.18 (0.68–2.05)	0.551	0.81 (0.57–1.14)	0.223	1.26 (0.72–2.21)	0.414
rs1983891	0.312	1.40 (1.01–1.94)	**0.046**	1.31 (0.78–2.19)	0.310	1.44 (1.03–2.00)	**0.034**	1.28 (0.76–2.15)	0.364
rs339331	**0.029**	1.33 (0.80–2.20)	0.266	1.57 (1.12–2.19)	**0.008**	1.30 (0.79–2.16)	0.307	1.51 (1.08–2.12)	**0.016**
rs6465657	0.423	-	-	1.10 (0.75–1.61)	0.628	-	-	1.06 (0.72–1.56)	0.780
rs1512268	0.563	1.18 (0.85–1.64)	0.336	1.24 (0.69–2.20)	0.472	1.22 (0.87–1.71)	0.245	1.30 (0.73–2.32)	0.380
rs12543663	0.631	1.25 (0.80–1.93)	0.326	-	-	1.32 (0.85–2.06)	0.215	-	-
rs10086908	**0.046**	0.38 (0.16–0.92)	**0.032**	1.06 (0.73–1.55)	0.754	0.38 (0.16–0.93)	**0.033**	1.07 (0.73–1.56)	0.740
rs1016343	0.081	1.08 (0.77–1.52)	0.658	1.62 (1.05–2.49)	**0.028**	1.08 (0.77–1.53)	0.647	1.65 (1.07–2.54)	**0.025**
rs13252298	0.559	0.92 (0.66–1.29)	0.619	1.26 (0.69–2.28)	0.455	0.90 (0.64–1.27)	0.55	1.36 (0.75–2.48)	0.313
rs6983561	0.138	1.21 (0.87–1.69)	0.259	1.82 (0.98–3.40)	0.059	1.22 (0.87–1.71)	0.241	1.88 (1.00–3.52)	0.048
rs16901966	0.052	1.28 (0.92–1.78)	0.142	2.18 (1.10–4.32)	**0.025**	1.28 (0.91–1.78)	0.153	2.26 (1.14–4.5)	**0.020**
rs16902094	0.675	1.16 (0.83–1.61)	0.386	1.15 (0.61–2.15)	0.669	1.18 (0.85–1.65)	0.327	1.18 (0.62–2.22)	0.619
rs445114	0.798	0.94 (0.63–1.41)	0.757	1.09 (0.76–1.56)	0.647	0.96 (0.64–1.44)	0.829	1.11 (0.77–1.6)	0.583
rs620861	0.976	0.96 (0.64–1.45)	0.849	1.01 (0.71–1.44)	0.966	0.97 (0.64–1.48)	0.894	1.02 (0.72–1.47)	0.895
rs6983267	0.297	1.33 (0.93–1.91)	0.120	1.12 (0.74–1.71)	0.591	1.27 (0.88–1.83)	0.196	1.11 (0.73–1.71)	0.622
rs1447295	**0.026**	1.58 (1.12–2.23)	**0.010**	-	-	1.59 (1.12–2.25)	**0.010**	-	-
rs11986220	**0.005**	1.80 (1.26–2.56)	**0.001**	-	-	1.76 (1.23–2.52)	**0.002**	-	-
rs10090154	**0.009**	1.74 (1.22–2.49)	**0.002**	-	-	1.71 (1.19–2.45)	**0.004**	-	-
rs7837688	0.176	1.39 (0.98–1.97)	0.063	1.27 (0.34–4.77)	0.725	1.36 (0.96–1.94)	0.084	1.27 (0.34–4.79)	0.727
rs10993994	0.587	1.23 (0.82–1.85)	0.315	1.13 (0.75–1.68)	0.566	1.21 (0.8–1.83)	0.363	1.11 (0.74–1.66)	0.624
rs7127900	**0.034**	-	-	0.56 (0.36–0.87)	**0.011**	-	-	0.54 (0.34–0.84)	**0.007**
rs7931342	**0.012**	0.72 (0.52–1.01)	0.054	1.65 (0.88–3.10)	0.120	0.73 (0.52–1.01)	0.059	1.72 (0.91–3.24)	0.094
rs9600079	0.636	1.12 (0.79–1.59)	0.514	1.19 (0.81–1.77)	0.378	1.08 (0.76–1.53)	0.681	1.17 (0.78–1.75)	0.443
rs4430796	**0.016**	0.75 (0.33–1.69)	0.483	1.69 (1.11–2.58)	**0.015**	0.73 (0.32–1.67)	0.457	1.66 (1.08–2.54)	**0.020**
rs1859962	0.306	1.2 (0.83–1.74)	0.322	1.39 (0.89–2.17)	0.153	1.20 (0.83–1.74)	0.334	1.43 (0.91–2.25)	0.118
rs2735839	0.081	0.63 (0.40–0.98)	0.042	1.04 (0.73–1.46)	0.844	0.62 (0.4–0.98)	0.042	1.01 (0.71–1.43)	0.954

Note: *p* < 0.05 are in bold. -The genetic models were not analyzed due to one of the genotype frequencies was less than 0.05.

### 3.3. GMDR Analyses Identified the Best Gene-Gene Interaction Model

**Figure 1:**
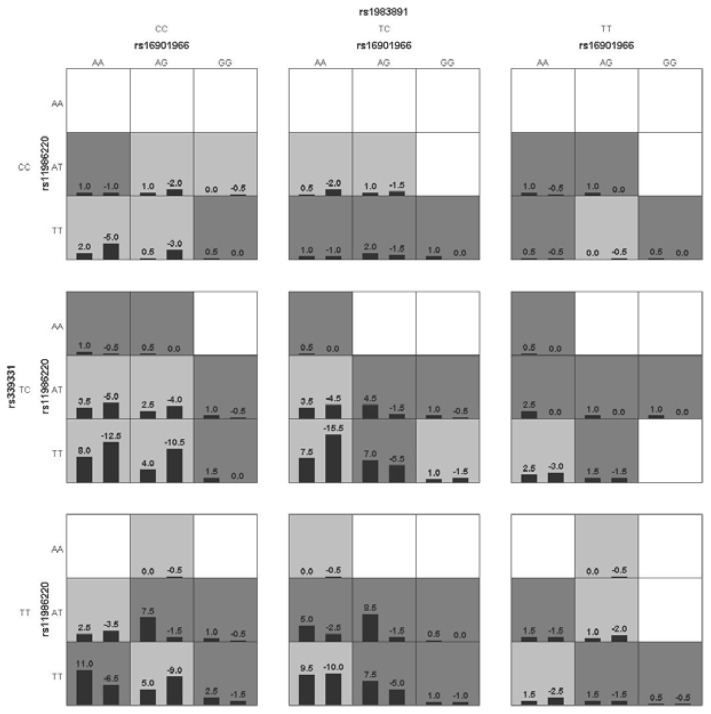
The best age-adjusted GMDR model for gene-gene interaction. The best model is composed of rs16901966, rs11986220, rs1983891, and rs339331. In each cell, the left bar represents a positive score, and the right bar a negative score. High-risk cells are indicated by dark shading, low-risk cells by light shading, and empty cells by no shading. The patterns of high-risk and low-risk cells differ across each of the different multilocus dimensions, presenting evidence of epistasis.

**Figure 2:**
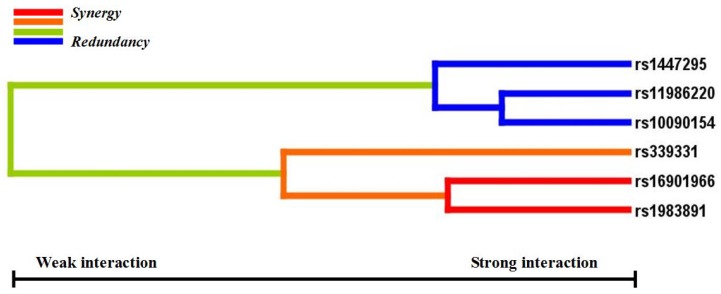
Gene-gene interaction dendrogram. The strongly interacting SNPs appear close together at the leaves of the tree (rs16901966 and 1983891), and the weakly interacting SNPs appear distant from each other.

Gene-gene interactions among rs16901966, rs11986220, rs1447295, rs10090154, rs1983891, and rs339331 showed that the sign test in four models had statistical significance (*p* < 0.05). However, only rs16901966, rs11986220, rs1983891, and rs339331 contributed to the best model with the smallest prediction error (1 − testing balanced accuracy [0.5785] = 0.4215) and the greatest CV consistency (10/10; sign test *p* = 0.001) (Table 4). Figure 1 shows the score distributions in the best model. The scores between case and control groups in different cells were different, indicating that patterns of high and low risk differ across each of the different multi-locus dimensions. This is evidence of gene-gene interactions. Entropy-based interaction dendrograms, built by MDR, showed the strongest synergy between rs1983891 at FOXP4 and rs16901966 at 8q24, followed by those two loci and rs339331 at RFX6 (Figure 2). The redundancy among rs11986220, rs10090154, and rs1447295 was consistent with their linkage degree [13].

**Table 4:** Age-adjusted GMDR models of gene-gene interactions among the six PCa associated SNPs.

Model	Training Bal.Acc.	Testing Bal.Acc.	Sign Test (*p*)	CV Consistency
rs11986220	0.5646	0.5556	9 (0.0107)	9/10
rs1983891 rs339331	0.5916	0.5329	8 (0.0547)	5/10
rs16901966 rs1983891 rs339331	0.6192	0.5626	8 (0.0547)	5/10
rs16901966 rs11986220 rs1983891 rs339331	0.6581	0.5785	10 (0.0010)	10/10
rs16901966 rs1447295 rs11986220 rs1983891 rs339331	0.6844	0.5690	9 (0.0107)	10/10
rs16901966 rs1447295 rs11986220 rs10090154 rs1983891 rs339331	0.6844	0.5663	9 (0.0107)	10/10

Note: Training Bal. ACC: Training Balanced Accuracy; Testing Bal. ACC: Testing Balanced Accuracy; CV: Cross Validation; The best model speculated by GMDR is composed of rs16901966, rs11986220, rs1983891 and rs339331.

### 3.4. Cumulative Effect of the SNPs that Increase the Risk of PCa

The cumulative effect of rs16901966, rs1447295, rs10090154, rs1983891, and rs339331 indicated that, compared to men without any of these risk variants, men who carried any combination of 1, 2, or ≥3 risk genotypes have a gradually increased risk of PCa. The OR (age-adjusted) was 4.14 (*p* = 2.22 × 10^−6^) (Table 5).

**Table 5:** Cumulative effects of risk variants on prostate cancer risk.

No. of Risk Genotypes *	Case	Control	Unadjusted OR		Age-Adjusted OR	
			OR (95% CI)	*p*	OR (95% CI)	*p*
0	26 (0.094)	55 (0.199)	1.00 (Reference)		1.00 (Reference)	
1	80 (0.290)	94 (0.339)	1.80 (1.04–3.13)	**0.036**	1.79 (1.02–3.12)	**0.042**
2	78 (0.284)	82 (0.296)	2.01 (1.15–3.52)	**0.014**	1.96 (1.11–3.46)	**0.020**
≥3	91 (0.331)	46 (0.166)	4.19 (2.33–7.52)	**9.050 × 10^−7^**	4.14 (2.30–7.46)	**2.220 × 10^−6^**

Note: * Risk genotypes were defined from the genetic model analysis of rs1983891 (TT, TC), rs339331 (TT), rs16901966 (GG), rs1447295 (AA, AC) and rs10090154 (TC, TT). *p* < 0.05 are in bold.

### 3.5. Logistic Regression Analyses for Independent Environmental Effects on SNPs

Results from case-only gene-environment interactions suggested that the ORs of PCa risk were increased when combined with some environments, compared with the independent SNPs association analysis (Table 6). Two genotype-BMI interactions were statistically significant: (1) rs6983561 CC with BMI ≥ 28 compared with rs6983561 CC with BMI < 24.0 (age-adjusted OR = 7.66; 95% CI: 1.20–49.04; *p* = 0.032); and (2) rs16901966 GG with BMI ≥ 28 compared with rs16901966 GG with BMI < 24.0 (age-adjusted OR = 5.33; 95% CI: 1.03–26.63; *p* = 0.046). In current smokers, carriers of rs7679673-C (CC + CA) and risk homozygous rs12653946 TT had increased ORs compared with rs7679673 AA and non-carriers with rs12653946 TC + CC (age-adjusted ORs = 2.77 and 3.12; 95% CI: 1.32–5.80 and 1.16–8.31; *p* = 0.007 and 0.024, respectively). The risk of PCa in often drinking cases with risk homozygous rs7679673 CC was 4.37-fold higher than often drinking cases with non-risk allelic carriers (rs7679673 CA + AA).

**Table 6:** The case-only study result of gene-environment interaction.

Environments × Gene			Unadjusted			Age-Adjusted		
			OR	95% CI	*p*	OR	95% CI	*p*
**BMI**	*rs6983561* CC	CA + AA						
<24.0	2 (0.036)	53 (0.964)	1 (Reference)			1 (Reference)		
24.0–<28.0	6 (0.107)	50 (0.893)	3.18	0.61–16.50	0.149	3.40	0.64–18.10	0.151
≥28	4 (0.250)	12 (0.750)	8.83	1.45–53.94	0.007	7.66	1.20–49.04	**0.032**
	*rs16901966* GG	GA + AA						
<24.0	3 (0.053)	54 (0.947)	1 (Reference)			1 (Reference)		
24.0–28.0	4 (0.068)	55 (0.932)	1.31	0.28–6.33	0.732	1.36	0.29–6.43	0.697
≥28	4 (0.250)	12 (0.750)	6.00	1.19–30.39	0.018	5.33	1.03–26.63	**0.046**
**Smoking**	*rs7679673* CC + CA	AA						
Never	21 (0.309)	47 (0.691)	1 (Reference)			1 (Reference)		
Current	32 (0.561)	25 (43.90)	2.87	1.38–5.97	0.004	2.77	1.32–5.80	**0.007**
	*rs12653946* TT	TC + CC						
Never	7 (0.100)	63 (0.900)	1 (Reference)			1 (Reference)		
Current	15 (0.259)	43 (0.741)	3.14	1.18–8.34	0.018	3.11	1.16–8.31	**0.024**
**Drinking**	*rs7679673* CC	CA + AA						
Seldom	5 (0.046)	103 (0.954)	1 (Reference)			1 (Reference)		
Often	3 (0.188)	13 (0.812)	4.75	1.07–22.25	0.032	4.37	1.27–20.09	**0.041**

Note: The risk allele of rs6983561, rs16901966, rs7679673, and rs12653946 are C, G, C, and T, respectively. Gene-environment interaction was analyzed by using the risk genotypes of SNPs.

## 4. Discussion

GWAS have identified over 70 loci associated with PCa. However, it is largely unknown which genes and environmental risk factors jointly affect the risk of this disease. The present study investigated the relationships between 36 GWAS identified PCa risk variants and the risk of PCa in a northern Chinese population. Based on allelic and genotypic association analyses, we explored the gene-gene interactions among six confirmed PCa risk variants and their cumulative effect on the risk of PCa in a case-control study. We also examined the gene-environment interaction between 36 SNPs and the environmental factors BMI, smoking, and alcohol consumption. The best gene-gene interaction model involved rs16901966 and rs11986220 at 8q24, rs1983891 at FOXP4, and rs339331 at RFX6. The cumulative effect of these six loci increased the risk of PCa about 3.2-fold. We observed that cases with BMI ≥ 28 had five to seven times higher risk of PCa with homozygous rs16901966 GG and rs6983561 CC than cases with BMI < 24.0. Cases that carried the risk allele C of rs7679673 (CC, CA) and homozygous rs7679673 CC, and were current smokers and often drinkers, had a 1.77 and 3.37-fold higher risk of PCa, respectively, compared to cases with identical genotypes but who never smoked and seldom drank. Current smokers with the rs12653946 TT genotype had 3.11 times the risk of PCa than never smokers.

After age adjustment, the confirmed PCa risk loci were rs16901966, rs11986220, rs1447295, and rs10090154 at 8q24, rs1983891 at FOXP4, and rs339331 at RFX6. In our previous study, rs16901966, rs1447295, rs11986220, and rs10090154 at 8q24 (Region 1, Region 2) were associated with PCa and PCa-related clinical covariates [13]. rs1983891 at FOXP4 and rs339331 at RFX6 were first identified as PCa risk loci in Japanese patients, and were further confirmed in subsequent studies [18,31,32]. Our result that only 6 of 36 SNPs were associated with PCa suggests that this discrepancy could result from gene-gene and gene-environment interactions that would alter the effect of some disease-associated genes or loci.

Gene-gene interaction analyses showed that there were significant joint interactions among rs16901966, and rs11986220 at 8q24, rs1983891 at FOXP4, and rs339331 at RFX6. rs16901966 (region 2), rs11986220 (region 1) at 8q24 on chromosome 8 were verified to have consistent associations in various populations [18,27,33,34,35]. rs1983891 is located in FOXP4, which belongs to subfamily P of the forkhead box (FOX) transcription factor family, and is associated with kidney tumors, larynx carcinoma, and breast tumors [36,37,38]. At present, the potential role for FOXP4 in prostate tumorigenesis has not been determined. rs339331 was located at RFX6, a member of the regulatory factor X (RFX) family of transcription factors. A recent study reported that rs339331 can affect the risk of PCa by altering the expression of RFX6 [39]. A family-based study reported an interaction between rs4242382 at 8q24 and rs10486567 at JAZF zinc finger1 gene (JAZF1), which encodes a transcriptional repressor, for non-aggressive PCa [40]. These two transcription factors, FOXP4 and RFX6, may also interact with 8q24 by some regulated loci.

GMDR can effectively eliminate noise from the covariate, can increase prediction accuracy, and is applicable to both dichotomous and continuous phenotypes in various population-based study designs [29]. The results of the GMDR analysis in our study established a biostatistical foundation for the study of functional epistasis between FOXP4, RFX6, and 8q24. To understand the cumulative effects of the confirmed six variants on the risk of PCa, we used a combined score of the total number of risk genotypes from the five SNPs that were independently associated with PCa (rs11986220 was excluded because of the linkage with rs10090154). Our data indicated that men who carried at least three risk genotypes had a three-fold increased risk of PCa compared with men who carried no risk genotypes. Because 33.1% of cases and 16.6% of control subjects carried three or more risk genotypes, the cumulative effects of these SNPs on PCa incidence in China are substantial. Furthermore, it has been verified that some variants at 8q24 are PCa-susceptible loci in different populations. Thus, confirming this cumulative effect in other populations is necessary.

PCa is a multifactorial process involving both genetic and environmental components. Studies on gene-environment interrelations can, therefore, provide a potentially powerful approach for identifying the causes of this disease [41]. Although the current work is a case-only study, analyses based only on cases are valid, and offer better precision for estimating gene-environment interactions than those based on full data [42]. Obesity, smoking, and alcohol consumption are associated with a moderate increase in the risk of PCa or aggressive PCa [43,44,45]. Gene-environment interactions could either decrease or increase the risk of the disease. However, which genes and factors jointly affect the risk of PCa is largely unknown. In our case-only study, we analyzed the interactions between 36 SNPs and BMI, smoking, and alcohol consumption. Our results showed that rs6983561 and rs16901966, that exhibit a high degree of linkage disequilibrium at 8q24, interacted with BMI ≥ 28 (obesity) and contributed to a higher risk of PCa (ORs = 7.66, 5.33, respectively). The homozygous and risk allele carriers of rs7679673 at TET2, that encode a protein involved in myelopoiesis, interact with smoking and drinking to increase the risk of PCa (ORs = 2.77, 4.37, respectively). Homozygous rs12653946 at 5p15 in current smokers can also increase the risk of PCa (OR = 3.11).

Although some significant risk factors were identified in our study, there are several limitations to the results. The largest limitation is the small sample size that lowers the statistical power of the study. Although the sample quality is higher, replicating these results in larger populations is desirable. Second, the small sample size and the number of SNP variants studied limited us to exploring the gene-gene interactions among only six confirmed PCa risk SNPs, thus ignoring some potential interactions among other SNPs. Third, the case-only study involved only 134 Beijing PCa cases, limiting the gene-environment-phenotype analysis (e.g., PSA level, Gleason score, tumor stage, and aggressive PCa). Furthermore, some valuable factors those associated with development PCa such as presence of diabetes or use of diabetic medication should be designed in the future study. The lack of family history or treatment methods (surgical operation or medications used) for adjustment is another major limitation of the analysis. Therefore, populations with detailed PCa-related environmental exposures should be examined in multiple centers of China to confirm the association between these SNPs-environment interactions and PCa. This would establish a PCa databank about gene-gene-phenotype and gene-environment-phenotype, and provide a more effective approach to evaluate the risk of PCa, and to provide recommendations for preventing this disease.

## 5. Conclusions

The results of the current case-control study demonstrated the effect of gene-gene interactions, and the cumulative effect among six PCa risk SNPs identified from 36 SNPs reported previously by GWAS, on the risk of PCa. These findings suggest an interaction exists among these six SNPs and that the cumulative effect can increase the risk of PCa. Additionally, a potential gene-environment interaction was found. The results indicated that several interactions between SNPs and BMI, smoking, or drinking contribute to the increased risk of PCa. Overall, these findings will be helpful for further epidemiological and functional investigations of the pathogenesis of PCa, as well as provide recommendations for preventing this disease.

## Supplementary Materials

The following are available online at www.mdpi.com/www.mdpi.com/1660-4601/13/2/162/s1, Table S1: PCR primers of 36 SNPs and annealing temperature for HRM, Table S2: PCR primers of 37 SNPs for sequencing, Table S3: Distribution of the risk alleles and genotypes (n/fre) of 36 SNPs in PCa cases and control subjects, and the test result for HWE.
