# Supplementary material for: The Cumulative Effect of Gene-Gene and Gene-Environment Interactions on the Risk of Prostate Cancer in Chinese Men
Source: Int J Environ Res Public Health. 2016 Jan 27;13(2):162. doi: 10.3390/ijerph13020162 (PMC4772182; doi:10.3390/ijerph13020162)
Supplement: Supplementary file 1 [file ijerph-13-00162-s001.pdf]

Supplementary Materials: The Cumulative Effect of Gene-Gene and Gene-Environment Interactions on the Risk of Prostate Cancer in Chinese Men

Ming Liu, Xiaohong Shi, Fan Yang, Jianye Wang, Yong Xu, Dong Wei, Kuo Yang, Yaoguang Zhang, Xin Wang, Siying Liang, Xin Chen, Liang Sun, Xiaoquan Zhu, Chengxiao Zhao, Ling Zhu, Lei Tang, Chenguang Zheng and Ze Yang

Table S1. PCR primers of 36 SNPs and annealing temperature for HRM.

SNPs	Primer (5′-3′)	Annealing Temperature
rs2710646F	5′-TTTAGGGGGACACAAGCA-3′	59 °C
rs2710646R	5′-GGACAGACAGATATCAAATACACTC-3′	
rs721048F	5′-CCTGAGGATTATCAAGGTCCC-3′	63 °C
rs721048R	5′-GGCTCCTGTTCCACACTG-3′	
rs1465618F	5′-TCTGGTAGAGCTTGGTAAGTCC-3′	59 °C
rs1465618R	5′-ATATTGTGAAGCCTTGGTG-3′	
rs12621278F	5′-TTCCTTGCTGCAGTAGACAGTGTT-3′	59 °C
rs12621278R	5′-CCAAGCAATTCATACCACTAAGACATC-3′	
rs2660753F	5′-AGGTACAGCAAGGGCAAACAA-3′	63 °C
rs2660753R	5′-GGGTTCGAATTGAGGGTCTAT-3′	
rs10934853F	5′-TTGGCCTGTGTGTTACA-3′	56 °C
rs10934853R	5′-GGCATACGCTTTGTG-3′	
rs17021918F	5′-GCTGCTATTTTTCCTT-3′	50 °C
rs17021918R	5′-TTACTAAGCAGGTGCTC-3′	
rs7679673F	5′-GCCAAGATTATAGGAA-3′	50 °C
rs7679673R	5′-TCATGGGAAAATTCTAC-3′	
rs9364554F	5′-CCACTATGATTAGTCCATCCTTGTC-3′	63 °C
rs9364554R	5′-ACAGCTCAAATGTGTTCACTC-3′	
rs6465657F	5′-TCTGGTACGTATTGGCTTA-3′	50 °C
rs6465657R	5′-CTCAAAGAGGAAGAAGTTAGG-3′	
rs1512268F	5′-CCCAATGCAGATTGAGTTA-3′	56 °C
rs1512268R	5′-GAAGCAAACCCTTTCTGTAGA-3′	
rs12543663F	5′-GCCACCTCAATATGAATA-3′	54 °C
rs12543663R	5′-CCATCAGCTTTTTAGTTTC-3′	
rs10086908F	5′-GATGAAATGAAACCAGTG-3′	51 °C
rs10086908R	5′-CTTCTATCACCTCAAACTT-3′	
rs1016343F	5′-CCAAGACCATGTGTTACA-3′	56 °C
rs1016343R	5′-GAACTGTGAAGCTGTGAGT-3′	
rs13252298F	5′-CTTGCTGTCTTCTCAGATA-3′	54 °C
rs13252298R	5′-CACACTTGCCATTTAG-3′	
rs6983561F	5′-AGGAGAATTGCAAACT-3′	54 °C
rs6983561R	5′-CCTTGATATGCAATTAGC-3′	
rs16901966F	5′-GGAAAACAGATGTCACAA-3′	54 °C
rs16901966R	5′-GAGGAAATGCTGGAAAAT-3′	
rs16902094F	5′-TAGATACAGGCCACCTTT-3′	54 °C
rs16902094R	5′-CCACAATAACCCAAATG-3′	
rs445114F	5′-CAAGCAGTGATCCGTTT-3′	50 °C
rs445114R	5′-GCAAGTAAGTGCTAAAATAAAGGTA-3′	
rs620861F	5′-TTGTAGTTGAATGCCTGTG-3′	50 °C
rs620861R	5′-AGCCCCTAAAATGGTAGATAC-3′	

Table S1. *Cont.*

SNPs	Primer (5′-3′)	Annealing Temperature
rs6983267F	5′-AGAGTTAATACCCTCATCGT-3′	50 °C
rs6983267R	5′-TTCTTTGTACTTTTCTCAGT-3′	
rs1447295F	5′-GCCATTGGGGAGGTATGT-3′	56 °C
rs1447295R	5′-GGGGTTCCTGTTGCTTT-3′	
rs10090154F	5′-CAGCTTTAATGCAATTCT-3′	50 °C
rs10090154R	5′-TCCAATATTGTTTTAGC-3′	
rs7837688F	5′-CCAACATTTCCCACTAGAG-3′	54 °C
rs7837688R	5′-GACGTGTCAACATAGACC-3′	
rs10993994F	5′-CTCAATGTGTTCTCACCTTGTTATC-3′	64 °C
rs10993994R	5′-TTTATACTGGAGAGGGCAACC-3′	
rs4962416F	5′-TGGAAGTTTCCTGACACCG-3′	59 °C
rs4962416R	5′-AGGAACAGGGAGTGACTCA-3′	
rs7127900F	5′-GGCCATGCGAGGAATGAT-3′	64 °C
rs7127900R	5′-GTGAGGCCTGTCCATGCT-3′	
rs7931342F	5′-ACAGGATCAGGGAAGTTGC-3′	59 °C
rs7931342R	5′-GCCCATCCTTCCACATTC-3′	
rs4430796F	5′-AGAGAGGCAGCACAGACT-3′	64 °C
rs4430796R	5′-GCCCTGCCCAATTTAAG-3′	
rs1859962F	5′-AGACTTTTCCAAATCCCTG-3′	54 °C
rs1859962R	5′-GCCCCATTATTAGAAATCTTG-3′	
rs2735839F	5′-CGCCTAGGGATCTGGTT-3′	56 °C
rs2735839R	5′-CCTCCTTCTTGGGATAGC-3′	
rs13385191F	5′-GCTGAGGTCCCTATGTC-3′	56 °C
rs13385191R	5′-CTCCAATCCTTCTTCTCG-3′	
rs12653946F	5′-CCACACAAATGTGCTTATG-3′	56 °C
rs12653946R	5′-CTTGCTTGTGATGAACAGA-3′	
rs1983891F	5′-GGTCCCATTCTCCTCGTA-3′	54 °C
rs1983891R	5′-ACAAGGACCAACAGACAG-3′	
rs339331F	5′-CACTTTGCATGAACTCTC-3′	51 °C
rs339331R	5′-CCTCCTAGTCACTAAAGATAAACCT-3′	
rs9600079F	5′-TTGCACCCACTTACAAT-3′	54 °C
rs9600079R	5′-TTGGTCTCAATTTTACAG-3′	

F: Forward primer; R: Reverse primer; Rs11986220 was genotyped by sequencing.

Table S2. PCR primers of 37 SNPs for sequencing.

SNPs	Primer (5′-3′)	Annealing Temperature
rs2710646F	5′-TTTAGGGGGACACAAGCA-3′	59 °C
rs2710646R	5′-GGACAGACAGATATCAAATACACTC-3′	
rs721048F	5′-CCTGAGGATTATCAAGGTCCC-3′	63 °C
rs721048R	5′-GGCTCCTGTTCCACACTG-3′	
rs1465618F	5′-TCTGGTAGAGCTTGGTAAGTCC-3′	59 °C
rs1465618R	5′-ATATTGTGAAGCCTTGGTG-3′	
rs12621278F	5′-TTCCTTGCTGCAGTAGACAGTGTT-3′	59 °C
rs12621278R	5′-CCAAGCAATTCATACCACTAAGACATC-3′	
rs2660753F	5′-AGGTACAGCAAGGGCAAACAA-3′	63 °C
rs2660753R	5′-GGGTTCGAATTGAGGGTCTAT-3′	

Table S2. *Cont.*

SNPs	Primer (5′-3′)	Annealing Temperature
rs10934853F<br>rs10934853R	5′-TTGGCCTGTGTGTTACA-3′<br>5′-GGCATACGCTTTGTG-3′	56 °C
rs17021918F<br>rs17021918R	5′-GCTGCTATTTTTCCTT-3′<br>5′-TTACTAAGCAGGTGCTC-3′	50 °C
rs7679673F<br>rs7679673R	5′-GCCAAGATTATAGGAA-3′<br>5′-TCATGGGAAAATTCTAC-3′	50 °C
rs9364554F<br>rs9364554R	5′-CCACTATGATTAGTCCATCCTTGTC-3′<br>5′-ACAGCTCAAATGTGTTCACTC-3′	63 °C
rs6465657F<br>rs6465657R	5′-TCTGGTACGTATTGGCTTA-3′<br>5′-CTCAAAGAGGAAGAAGTTAGG-3′	50 °C
rs1512268F<br>rs1512268R	5′-CCCAATGCAGATTGAGTTA-3′<br>5′-GAAGCAAACCCTTTCTGTAGA-3′	56 °C
rs12543663F<br>rs12543663R	5′-GCCACCTCAATATGAATA-3′<br>5′-CCATCAGCTTTTTAGTTTC-3′	54 °C
rs10086908F<br>rs10086908R	5′-GATGAAATGAAACCAGTG-3′<br>5′-CTTCTATCACCTCAAACTT-3′	51 °C
rs1016343F<br>rs1016343R	5′-CCAAGACCATGTGTTACA-3′<br>5′-GAACTGTGAAGCTGTGAGT-3′	56 °C
rs13252298F<br>rs13252298R	5′-CTTGCTGTCTTCTCAGATA-3′<br>5′-CACACTTGCCATTTAG-3′	54 °C
rs6983561F<br>rs6983561R	5′-AGGAGAATTGCAAACT-3′<br>5′-CCTTGATATGCAATTAGC-3′	54 °C
rs16901966F<br>rs16901966R	5′-GGAAAACAGATGTCACAA-3′<br>5′-GAGGAAATGCTGGAAAAT-3′	54 °C
rs16902094F<br>rs16902094R	5′-TAGATACAGGCCACCTTT-3′<br>5′-CCACAATAACCCAAATG-3′	54 °C
rs445114F<br>rs445114R	5′-CAAGCAGTGATCCGTTT-3′<br>5′-GCAAGTAAGTGCTAAAATAAAGGTA-3′	50 °C
rs620861F<br>rs620861R	5′-TTGTAGTTGAATGCCTGTG-3′<br>5′-AGCCCCTAAAATGGTAGATAC-3′	50 °C
rs6983267F<br>rs6983267R	5′-AGAGTTAATACCCTCATCGT-3′<br>5′-TTCTTTGTACTTTTCTCAGT-3′	50 °C
rs1447295F<br>rs1447295R	5′-GCCATTGGGGAGGTATGT-3′<br>5′-GGGGTTCCTGTTGCTTT-3′	56 °C
rs10090154F<br>rs10090154R	5′-CAGCTTTAATGCAATTCT-3′<br>5′-TCCAATATTGTTTTAGC-3′	50 °C
rs7837688F<br>rs7837688R	5′-CCAACATTTCCCACTAGAG-3′<br>5′-GACGTGTCAACATAGACC-3′	54 °C
rs10993994F<br>rs10993994R	5′-CTCAATGTGTTCTCACCTTGTTATC-3′<br>5′-TTTATACTGGAGAGGGCAACC-3′	64 °C
rs4962416F<br>rs4962416R	5′-TGGAAGTTTCCTGACACCG-3′<br>5′-AGGAACAGGGAGTGACTCA-3′	59 °C
rs7127900F<br>rs7127900R	5′-GGCCATGCGAGGAATGAT-3′<br>5′-GTGAGGCCTGTCCATGCT-3′	64 °C
rs7931342F<br>rs7931342R	5′-ACAGGATCAGGGAAGTTGC-3′<br>5′-GCCCATCCTTCCACATTC-3′	59 °C
rs4430796F<br>rs4430796R	5′-AGAGAGGCAGCACAGACT-3′<br>5′-GCCCTGCCCAATTTAAG-3′	64 °C

Table S2. *Cont.*

SNPs	Primer (5′-3′)	Annealing Temperature
rs1859962F	5′-AGACTTTTCCAAATCCCTG-3′	54 °C
rs1859962R	5′-GCCCCATTATTAGAAATCTTG-3′	
rs2735839F	5′-CGCCTAGGGATCTGGTT-3′	56 °C
rs2735839R	5′-CCTCCTTCTTGGGATAGC-3′	
rs13385191F	5′-GCTGAGGTCCCTATGTC-3′	56 °C
rs13385191R	5′-CTCCAATCCTTCTTCTCG-3′	
rs12653946F	5′-CCACACAAATGTGCTTATG-3′	56 °C
rs12653946R	5′-CTTGCTTGTGATGAACAGA-3′	
rs1983891F	5′-GGTCCCATTCTCCTCGTA-3′	54 °C
rs1983891R	5′-ACAAGGACCAACAGACAG-3′	
rs339331F	5′-CACTTTGCATGAACTCTC-3′	51 °C
rs339331R	5′-CCTCCTAGTCACTAAAGATAAACCT-3′	
rs9600079F	5′-TTGCACCCACTTACAAT-3′	54 °C
rs9600079R	5′-TTGGTCTCAATTTTACAG-3′	
rs11986220F	5′-ACCATGTTTAACTAACCCAC-3′	57 °C
rs11986220R	5′-CTTATAGCTTACGACCTTACTG-3′	

F: Forward primer; R: Reverse primer.

Table S3. Distribution of the risk alleles and genotypes (n/fre) of 36 SNPs in PCa cases and control subjects, and the test result for HWE.

SNPs ID	Our Study										Hapmap
	Alleles	Allele1 (n/RAF)			Genotype (n/freq)						
	(1/2)	Case	Control	HWE	Case			Control			RAF
		1	1	(*p*)	11	12	22	11	12	22	
rs721048	**A**/G	20 (0.038)	12 (0.024)	0.697	2 (0.008)	16 (0.061)	244 (0.931)	0 (0.000)	12 (0.048)	237 (0.952)	0.020
rs2710646	**A**/C	24 (0.042)	20 (0.035)	0.000	3 (0.010)	18 (0.062)	268 (0.927)	3 (0.010)	14 (0.049)	269 (0.941)	0.020
rs1465618	**A**/G	391 (0.694)	350 (0.646)	0.505	138 (0.496)	110 (0.396)	30 (0.108)	118 (0.445)	114 (0.430)	33 (0.125)	0.670
rs12621278	**A**/G	406 (0.720)	411 (0.719)	0.920	148 (0.525)	110 (0.390)	24 (0.085)	148 (0.517)	115 (0.402)	23 (0.080)	0.760
rs13385191	**G**/A	272 (0.472)	255 (0.444)	0.384	68 (0.236)	136 (0.472)	84 (0.292)	53 (0.185)	149 (0.519)	85 (0.296)	0.430
rs2660753	**A**/G	186 (0.338)	171 (0.330)	0.828	34 (0.124)	118 (0.429)	123 (0.447)	29 (0.112)	113 (0.436)	117 (0.452)	0.360
rs10934853	**A**/C	243 (0.423)	247 (0.432)	0.937	54 (0.188)	135 (0.470)	98 (0.341)	53 (0.185)	141 (0.493)	92 (0.322)	0.420
rs17021918	**C**/T	373 (0.650)	379 (0.667)	0.138	119 (0.415)	135 (0.470)	33 (0.115)	132 (0.465)	115 (0.405)	37 (0.130)	0.680
rs7679673	**C**/A	124 (0.225)	117 (0.210)	0.413	19 (0.069	86 (0.313)	170 (0.618)	10 (0.036)	97 (0.348)	172 (0.616)	0.160
rs12653946	**T**/C	249 (0.431)	229 (0.398)	0.386	58 (0.201)	133 (0.460)	98 (0.339)	42 (0.146)	145 (0.503)	101 (0.351)	0.330
rs9364554	**T**/C	178 (0.327)	181 (0.342)	0.180	31 (0.114)	116 (0.426)	125 (0.460)	26 (0.098)	129 (0.487)	110 (0.415)	0.360
rs1983891	**T**/C	205 (0.355)	180 (0.314)	0.626	36 (0.125)	133 (0.460)	120 (0.415)	30 (0.105)	120 (0.418)	137 (0.477)	0.410
rs339331	**T**/C	399 (0.690)	351 (0.620)	0.643	141 (0.488)	117 (0.405)	31 (0.107)	107 (0.378)	137 (0.484)	39 (0.138)	0.690
rs6465657	**C**/T	506 (0.878)	490 (0.863)	0.410	221 (0.767)	64 (0.222)	3 (0.010)	213 (0.750)	64 (0.225)	7 (0.025)	0.910
rs1512268	**A**/G	196 (0.340)	179 (0.312)	0.177	28 (0.097)	140 (0.486)	120 (0.417)	23 (0.080)	133 (0.463)	131 (0.456)	0.340
rs12543663	**C**/A	58 (0.101)	48 (0.084)	0.127	6 (0.021)	46 (0.160)	235 (0.819)	4 (0.014)	40 (0.140)	242 (0.846)	0.110
rs10086908	**T**/C	412 (0.786)	347 (0.803)	0.558	171 (0.653)	70 (0.267)	21 (0.080)	138 (0.639)	71 (0.329)	7 (0.032)	0.790
rs1016343	**T**/C	246 (0.429)	218 (0.384)	0.967	63 (0.220)	120 (0.418)	104 (0.362)	42 (0.148)	134 (0.472)	108 (0.380)	0.400
rs13252298	**G**/A	159 (0.285)	153 (0.287)	0.783	27 (0.097)	105 (0.376)	147 (0.527)	21 (0.079)	111 (0.416)	135 (0.506)	0.330
rs6983561	**C**/A	167 (0.300)	144 (0.254)	0.680	29 (0.104)	109 (0.392)	140 (0.504)	17 (0.060)	110 (0.389)	156 (0.551)	0.290

Table S3. *Cont.*

SNPs ID	Our Study										Hapmap
	Alleles	Allele1 (n/RAF)			Genotype (n/freq)						
	(1/2)	Case	Control	HWE	Case			Control			RAF
		1	1	(*p*)	11	12	22	11	12	22	
rs16901966	**G**/A	171 (0.299)	139 (0.244)	0.205	27 (0.094)	117 (0.409)	142 (0.497)	13 (0.046)	113 (0.396)	159 (0.558)	0.290
rs16902094	**G**/A	160 (0.288)	152 (0.265)	0.970	22 (0.079)	116 (0.417)	140 (0.504)	20 (0.070)	112 (0.390)	155 (0.540)	0.310
rs445114	**T**/C	311 (0.546)	309 (0.542)	0.675	87 (0.305)	137 (0.481)	61 (0.214)	82 (0.288)	145 (0.509)	58 (0.204)	0.510
rs620861	**C**/T	320 (0.559)	309 (0.562)	0.769	92 (0.322)	136 (0.476)	58 (0.203)	88 (0.320)	133 (0.484)	54 (0.196)	0.600
rs6983267	**G**/T	261 (0.463)	239 (0.424)	0.930	56 (0.199)	149 (0.528)	77 (0.273)	51 (0.181)	137 (0.486)	94 (0.333)	0.390
rs1447295	**A**/C	124 (0.224)	94 (0.164)	0.111	8 (0.029)	108 (0.390)	161 (0.581)	4 (0.014)	86 (0.300)	197 (0.686)	0.110
rs11986220	**A**/T	118 (0.211)	81 (0.145)	0.370	5 (0.018)	108 (0.387)	166 (0.595)	4 (0.014)	73 (0.261)	203 (0.725)	0.100
rs10090154	**T**/C	116 (0.208)	81 (0.145)	0.370	5 (0.018)	106 (0.380)	168 (0.602)	4 (0.014)	73 (0.261)	203 (0.725)	0.100
rs7837688	**T**/C	113 (0.203)	92 (0.163)	0.126	5 (0.018)	103 (0.369)	171 (0.613)	4 (0.014)	84 (0.298)	194 (0.688)	0.100
rs10993994	**T**/C	280 (0.522)	218 (0.491)	0.350	75 (0.280)	130 (0.485)	63 (0.235)	57 (0.257)	104 (0.468)	61 (0.275)	0.410
rs7127900	**G**/A	464 (0.847)	352 (0.900)	0.403	195 (0.712)	74 (0.270)	5 (0.018)	159 (0.815)	33 (0.169)	3 (0.015)	0.870
rs7931342	**G**/T	149 (0.260)	162 (0.283)	0.084	27 (0.094)	95 (0.332)	164 (0.573)	17 (0.059)	128 (0.448)	141 (0.493)	0.230
rs9600079	**T**/G	268 (0.464)	251 (0.436)	0.302	68 (0.235)	132 (0.457)	89 (0.308)	89 (0.308)	133 (0.462)	96 (0.333)	0.500
rs4430796	**A**/G	298 (0.764)	227 (0.709)	0.178	119 (0.610)	60 (0.308)	16 (0.082)	77 (0.481)	73 (0.456)	10 (0.062)	0.710
rs1859962	**G**/T	242 (0.453)	217 (0.409)	0.384	54 (0.202)	134 (0.502)	79 (0.296)	41 (0.155)	135 (0.509)	89 (0.336)	0.490
rs2735839	**G**/A	330 (0.579)	340 (0.607)	0.191	102 (0.358)	126 (0.442)	57 (0.200)	98 (0.350)	144 (0.514)	38 (0.136)	0.640

The risk alleles determined in our study are in bold. 1 and 2 represent the risk and non-risk alleles, respectively. 11, 12 and 22 represent the allele corresponding genotypes. SNP: single nucleotide polymorphism; RAF: risk allele frequency; HWE: Hardy-Weinberg equilibrium.

© 2016 by the authors; licensee MDPI, Basel, Switzerland. This article is an open access article distributed under the terms and conditions of the Creative Commons by Attribution (CC-BY) license (http://creativecommons.org/licenses/by/4.0/).
